# Folding a focalized acoustical vortex on a flat holographic transducer: Miniaturized selective acoustical tweezers

**DOI:** 10.1126/sciadv.aav1967

**Published:** 2019-04-12

**Authors:** Michael Baudoin, Jean-Claude Gerbedoen, Antoine Riaud, Olivier Bou Matar, Nikolay Smagin, Jean-Louis Thomas

**Affiliations:** 1 Université de Lille, CNRS, Centrale Lille, ISEN, Université Polytechnique des Hauts-de-France, UMR 8520, SATT Nord, IEMN, International laboratory LIA/LICS, F-59000 Lille, France.; 2 Université Paris Sorbonne Cité, INSERM UMR-S1147, 45 Rue des Saints Pères, 75270 Paris, France.; 3 Sorbonne Universités, UPMC Université Paris 06, CNRS, UMR 7588, Institut des NanoSciences de Paris, 4 Place Jussieu, 75005 Paris, France.

## Abstract

Acoustical tweezers based on focalized acoustical vortices hold the promise of precise contactless manipulation of millimeter down to submicrometer particles, microorganisms, and cells with unprecedented combined selectivity and trapping force. Yet, the widespread dissemination of this technology has been hindered by severe limitations of current systems in terms of performance and/or miniaturization and integrability. Here, we unleash the potential of focalized acoustical vortices by developing the first flat, compact, paired single electrode focalized acoustical tweezers. These tweezers rely on spiraling transducers obtained by folding a spherical acoustical vortex on a flat piezoelectric substrate. We demonstrate the ability of these tweezers to grab and displace micrometric objects in a standard microfluidic environment with unique selectivity. The simplicity of this system and its scalability to higher frequencies open tremendous perspectives in microbiology, microrobotics, and microscopy.

## Teaser

Selective manipulation of micrometric objects in a standard microscopy environment is possible with miniaturized acoustical tweezers.

## INTRODUCTION

The precise contactless manipulation of physical and biological objects at micrometric down to nanometric scales promises tremendous development in fields as diverse as microrobotics, tissue engineering, or micro/nanomedicine. In this regard, acoustical tweezers are a prominent technology since they are noninvasive, biocompatible (*1*–*3*), and label free and enable trapping forces several orders of magnitudes larger than their optical counterparts at same actuation power (*4*, *5*). The first reported observations of particle levitation in acoustic wave fields date back to the work of Boyle and Lehmann (*6*). Nevertheless, only recent simultaneous developments of advanced wave synthesis systems, microfluidic setups, and theory of acoustic radiation pressure enabled harnessing the potential of acoustophoresis (*5*, *7*–*19*). Until recently, the vast majority of acoustical tweezers relied on a single or a set of orthogonal standing waves that create a network of nodes and antinodes where particles are trapped (*7*–*12*, *14*, *16*–*18*). These systems are highly efficient for the collective manipulation of particles and cells, but the multiplicity of traps and the agglomeration of several particles at the same node or antinode preclude any selectivity. While limited localization of the acoustic energy can be achieved by the original sub–time-of-flight techniques (*20*), only strong focalization of wave fields enables selectivity at the single particle level. Focalized acoustic waves are thus natural candidates to achieve this localization (*21*–*23*), but many particles of practical interest migrate to the nodes of standing wave fields such as rigid particles and cells (*24*, *25*) and are thus expelled from the wave focus. This radial expelling and the condition to get a restoring axial force are the main difficulties that thwarted research on selective acoustical tweezers. The combination of strong localization and the existence of a minimum of the pressure wave field at the focus point surrounded by a bright ring is fulfilled by so-called cylindrical or spherical acoustical vortices, some helical waves spinning around a phase singularity (*26*–*29*). These waves are the separate variable solutions of Helmholtz equation in cylindrical and spherical coordinates, respectively. The former are invariant in the *z* direction and thus enable only two-dimensional (2D) particle trapping, while focalized spherical vortices enable 3D particle trapping with a single beam, as first demonstrated theoretically by Baresch *et al*. (*4*, *29*), following recent developments in the theory of acoustic radiation force (*30*, *31*). A wealth of systems (*5*, *13*, *15*, *19*, *28*, *32*–*37*) have been proposed since the seminal work by Hefner and Marston (*27*) for the synthesis of acoustical vortices. Nevertheless, the ability to obtain a 3D trap and to pick up one particle independently of its neighbors was only demonstrated recently by Baresch *et al*. (*5*).This operation requires a strong focalization of the acoustical vortex (*29*). In addition, all acoustical vortices synthesis systems developed to date rely on either arrays of transducers (*5*, *13*, *15*, *28*, *32*, *33*) or passive systems (*35*–*37*) that are cumbersome, hardly miniaturizable, and incompatible with microscopes or microfluidics chips. Recently, Riaud *et al*. (*19*) showed that it is possible to generate cylindrical acoustical vortices with swirling surface acoustic waves (SAWs) synthesized with spiraling interdigitated transducers (IDTs) printed at the surface of a piezoelectric substrate. This system is flat, transparent, and compatible with disposable substrates, and its fabrication with standard lithography is straightforward to miniaturize and is cheap as proved by its widespread use in modern electronic devices (*38*). This wave synthesis system nevertheless suffers from major limitations as follows: (i) Lateral focalization of cylindrical acoustical vortices is weaker than 3D focalization of their spherical counterparts and leads to the existence of spurious secondary rings of weaker amplitude that can also trap particles, hence severely limiting the tweezers selectivity; (ii) the anisotropy of piezoelectric substrates leads to an anisotropy of surface waves and, hence, of the trapping force; and (iii) 3D trapping is not possible with cylindrical acoustical vortices. In summary, the swirling SAW technology has come with its own set of challenges and limitations, and a paradigm shift is needed to reach true selectivity without compromising on miniaturization.

Here, we harness the potential of selective acoustical tweezers by folding the phase of a focalized acoustical vortex on a flat surface following the principle of Fresnel lenses and synthesize it with single spiraling interdigitated electrodes deposited at the surface of a piezoelectric substrate. These electrodes materialize two equiphase lines, hence discretizing the folded phase on two levels. The shape of the electrodes is reminiscent of an Archimedes-Fermat spiral, an intermediate situation between the Archimedes spiral (linear dependency of the radius over the angle) and the Fermat spiral (quadratic dependency). The radial contraction of the spiral enables wave focusing without the requirement of a curved transducer or a lens, a major advantage compared to existing systems. In addition, all the limitations of the cylindrical vortex–based tweezers are overcome. We demonstrate the high selectivity of this tweezer (i) by measuring the acoustic field with a laser interferometer and quantifying the fast radial decrease of secondary rings and (ii) by selectively trapping and moving one particle independently of its neighbors in a standard microfluidic environment.

## MATERIALS AND METHODS

### Theory: Folding a spherical acoustical vortex on a flat surface

Spherical acoustical Bessel beams (spherical vortices) constitute excellent candidates to create a localized acoustic trap. These acoustic fields both focalize the acoustic energy in 3D and create a shadow zone at the center of the vortex surrounded by a bright shell, wherein particles can be trapped (*29*). In the same way that a plane standing wave can be seen as the combination of two counterpropagating traveling waves, a spherical Bessel beam results from the interference between a converging and a diverging spherical Hankel beam (of the first and second kinds, respectively). Hence, a Bessel beam can be produced by a single Hankel converging beam, which will interfere with its diverging counterpart generated at the focus, i.e., at the vortex central singularity. Baresch *et al*. (*29*) demonstrated that the result for a one-sided source of finite aperture is a composition of Hankel and Bessel beams, with the Bessel contribution vanishing in the far field. This is why it is only necessary to consider the Hankel contribution for the source in the far-field approximation.

**Fig. 1:**
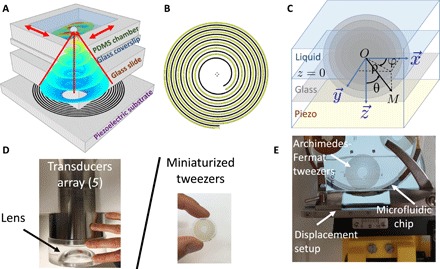
Archimedes-Fermat acoustical tweezers: Principle. (**A**) Scheme illustrating the composition of the Archimedes-Fermat acoustical tweezers: A focalized acoustical vortex is synthesized by spiraling metallic electrodes deposited at the surface of a piezoelectric substrate. The vortex propagates and focalizes inside a glass slide (sealed with the piezoelectric substrate) and a mobile glass coverslip before reaching the liquid contained in a polydimethylsiloxane (PDMS) chamber, wherein the particle is trapped. The mobility of the microfluidic chip (glass coverslip and sealed PDMS chamber) is enabled by a liquid couplant and a manual precision displacement setup represented in (E). (**B**) Spiraling pattern of the electrodes obtained from approximated Eq. 2 with $\tilde{z}=55.4$ and *m* = 1. (**C**) Scheme introducing the spherical (*r*, θ, φ) and cylindrical coordinates (ρ, φ, *z*) used for the demonstration of Eq. 2. (**D**) Comparison of the compactness of the transducer array of (*5*) (left) to the Archimedes-Fermat acoustical tweezers presented in this paper (right). This figure also shows the transparency of the Archimedes-Fermat acoustical tweezers (particles are trapped on the central axis of the transducer). Photo credit: Jean-Louis Thomas, CNRS (left) and Michael Baudoin, Université de Lille (right). (**E**) Image showing the integration of the Archimedes-Fermat acoustical tweezers into a Leica Z16 macroscope. Four tweezers have been patterned on a 3-inch LiNbO_3_ wafer. Photo credit: Jean-Claude Gerbedoen, SATT Nord.

Here, we demonstrated that converging Hankel beams of finite aperture can be synthesized by materializing the intersections between the Hankel beam isophase surfaces (which can be visualized on Fig. 1A) and a plane, with metallic electrodes deposited at the surface of a piezoelectric substrate. Each electrode will then be excited electrically with the corresponding phase, hence provoking localized vibrations of the piezoelectric substrate, which in turn produce a bulk acoustical vortex inside a glass slide. This original holographic method combines the underlying physical principles of Fresnel lenses in optics (wherein an isophase is folded on a flat surface), the specificity of Bessel beam topology, and the principles of wave synthesis with IDTs in the field of microelectronics.

The general expression of a Hankel spherical vortex in the complex plane is given by

$$\Psi *(r,\theta ,\varphi )=Ah_{l}(kr)P_{l}^{m}(\text{cos}(\theta ))e^{i(-m\varphi +\omega t)}$$

with (*r*, θ, φ) as the spherical coordinates, *k* = ω/*c*_*a*_ as the wave number, *c*_*a*_ as the sound speed, *A* > 0 as the wave amplitude, $P_{l}^{m}$ as the Legendre polynomial of order (*l*, *m*), ω, as the frequency, *t* as the time, and *h*_*l*_ as the spherical Hankel function of the first kind. To compute the intersection of the Hankel beam with a plane, we introduce the cylindrical coordinates (ρ, φ, *z*), with $r=\sqrt{\rho^{2}+z^{2}}$ and *z* as the axis normal to the plane (see Fig. 1C). For the sake of simplicity, we also introduced the dimensionless parameters $\bar{r}=kr$, $\bar{\rho }=k\rho$, and $\bar{z}=kz$. In the far field, ($\bar{z}>>1$ and thus $\bar{r}>>1$), Hankel functions of the first kind can be approximated by $h_{l}(\bar{r})={\left(-i\right)}^{l+1}e^{i\bar{r}}/\bar{r}$, and thus, isophases of Ψ* are simply given by the equation

$$\begin{array}{c} \varphi =\;\text{arg}(\Psi *)=\;\text{arg}(h_{l}(\bar{r}))+\text{arg}(e^{i(-m\varphi +\omega t)})\\ \approx \bar{r}-(l+1)\frac{\pi }{2}-m\varphi +\omega t=C_{1} \end{array}$$ (1)

with *C*_1_ a constant. Since *t* and the constant (left hand side) were arbitrarily chosen, this equation reduces to the simple expression $\varphi =\bar{r}-m\varphi =C_{2}$, with $\bar{r}=\sqrt{{\bar{\rho }}^{2}+{\bar{z}}^{2}}$ and *C*_2_ a constant.

Because of the piezoelectric effect, the mechanical vibrations of the bulk acoustic waves were coupled to the electrical potential. Following the principle of wave synthesis with IDTs, we modeled the electrodes as perfect wires (isopotential lines). If we consider two spirals in opposition of phase (Δφ = π), we obtain the following polar equations of the two metallic tracks

$${\bar{\rho }}_{1}=\sqrt{{\left(m\varphi +C_{2}\right)}^{2}-{\bar{z}}^{2}}\;\text{and}\;{\bar{\rho }}_{2}=\sqrt{{\left(m\varphi +C_{2}+\pi \right)}^{2}-{\bar{z}}^{2}}$$ (2)

With these two electrodes, the folded phase was discretized on two levels.

These simple equations correspond to spirals, as represented in Fig. 1B (with $\bar{z}=55.4$ and *m* = 1). It is an intermediate situation between the Fermat spiral ($\bar{\rho }$∝φ^2^) and the Archimedes spiral ($\bar{\rho }$∝φ). The radial distance between consecutive electrodes decreases progressively (as shown by the second derivative of these equations), and the spirals converge asymptotically toward Archimedes arithmetic spirals of equation $\bar{\rho }=m\varphi +K$ (with *K* = 0 or π). The archimedes spiral equation corresponds to the equiphase lines of a cylindrical vortex. This equation cannot ensure the focalization of the wave. This is therefore the geometric regression of the radial distance between consecutive tracks, which produces the beam focusing. We can note that Eq. 2 is only a far-field approximation of the exact solution of the problem. The exact shape of the spirals can be obtained by directly solving the nonlinear Eq. 1. We numerically solved this equation with an interior-point method and observed that the approximate and exact spirals only differ in the vicinity of the spiral center. Since the center of the spiral was not materialized in the present tweezers (to leave space for visualization), the exact and approximated shape of the electrodes matched perfectly (see fig. S1), and this difference did not affect the synthesized wave field.

### Experimental design of the tweezer

Experimentally, a system was designed to synthesize focalized vortices of topological order (*l*, *m*) = (1, 1) at frequency *f* = ω/2π = 4.4 MHz. This system relies on spiraling metallic electrodes deposited at the surface of a 0.5-mm-thick Y-36 niobate lithium (LiNbO_3_) piezoelectric substrate, following a standard lift-off procedure as follows: (i) A negative mask with the electrodes represented in Fig. 1B was obtained with high-resolution printing, (ii) a sacrificial layer (Photoresist AZnLOF 2020) with a thickness of 3 μm was deposited on the substrate and patterned using conventional photholithography technique, (iii) a titanium (Ti) layer of 20 nm and a gold (Au) layer of 200 nm were successively evaporated on the LiNbO_3_ substrate, and (iv) the sacrificial layer was washed out with *N*-methyl-2-pyrrolidone–based solvent stripper. The vibration of these spiraling electrodes driven by a Tektronix AFG 3051C waveform generator and an AR150A250 amplifier instilled a converging Hankel beam inside a Borofloat glass slide with a thickness of 6.5 mm glued on the surface of the piezoelectric substrate with EPO-TEK 301-2 (see Fig. 1A). The properties of the glass slide were chosen to match the acoustic properties of a mobile Borofloat glass coverslip with a thickness of 150 μm, supporting a polydimethylsiloxane (PDMS) channel with a depth of 300 μm glued with O_2_ plasma. The microfluidic chip (glass coverslip and PDMS chamber) containing the particles was coupled with the glass slide with a drop of oil and moved with a manual precision displacement setup (Fig. 1E). The electrodes were designed (i) to generate transverse waves of speed *C*_T_ into the glass slide, (ii) to obtain a focalization at the surface of the coverslip and thus enable optimal wave localization in the microfluidic chamber, and (iii) to obtain a transducer aperture of 64°. Transverse waves in the glass were chosen, owing to their lower sound speed (*C*_T_ = 3280 ms^−1^), which ensures better transmission of the acoustic energy from the glass to the liquid. The resulting acoustic field at the surface of the glass coverslip (focal plane, *z* = 0) was measured with a UHF-120 Polytec laser vibrometer.

### Numerical prediction of the resulting acoustic field

Predictions of the acoustic field expected at the surface of the glass coverslip (in contact with the liquid) were obtained by propagating the vibrations produced by the spiraling transducers in the glass slide and coverslip with an angular spectrum code (*39*). This latter method relies on (i) the 2D spatial Fourier transform of a wave field in a source plane (electrode plane), (ii) the propagation of each corresponding plane wave in the Fourier space to a target plane, and (iii) the inverse Fourier transform of the result.

## RESULTS

**Fig. 2:**
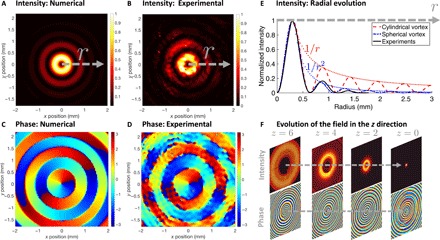
Field synthesized by an Archimedes-Fermat acoustical tweezers: Theory versus experiments. (**A**) Numerical predictions with the angular spectrum method and (**B**) experimental measurements with a UHF-120 Polytec laser interferometer of the normalized intensity of the vibration at the surface of the glass coverslip (focal plane, *z* = 0). The maximum amplitude measured experimentally (on the first ring) is 10 nm. (**C**) Numerical predictions with the angular spectrum method and (**D**) experimental measurements with the laser interferometer of the phase of the acoustic wave at the surface of the glass coverslip. (**E**) Radial evolution of the normalized intensity of the acoustic wave from the center of the vortex to the side, as a function of the lateral radius *r* in millimeters. Black solid line: Average over all angles φ of the intensity measured experimentally. Red dashed line: Evolution expected for a cylindrical vortex (cylindrical Bessel function). Blue dashed-dotted line: Evolution expected for a spherical vortex (spherical Bessel function). Red dotted line: Asymptotic evolution in 1/*r*. Blue dotted line: Asymptotic evolution in 1/*r*^2^. (**F**) Evolution of the field intensity (top) and phase (bottom) in the *z* direction. The direction of the arrow indicates the wave propagation direction. Left to right: Distances *z* = 6, 4, 2, and 0 mm, respectively (*z* = 0 corresponds to the focal plane). Top: Localization of the acoustic energy and formation of a localized trap. Bottom: Transition from a Hankel to a Bessel spherical beam.

We first compared the acoustic field measured experimentally to the numerical predictions obtained from the angular spectrum method (see Fig. 2, A to D and movie S1). An excellent agreement between the numerical predictions and the experiments is obtained for both the intensity (Fig. 2, A and B) and the phase (Fig. 2, C and D) of the wave field. In addition, the radial evolution of the rings’ intensity [in the (*x*, *y*) plane] measured experimentally and averaged over all angles ρ (black continuous line) is compared to (i) the radial evolution of a cylindrical vortex (red dashed line) and (ii) the radial evolution of a spherical vortex (blue dashed-dotted line), as shown in Fig. 2E. This figure shows (i) that the radial evolution of the vortex intensity measured experimentally closely follows the one of a spherical vortex and (ii) that the intensity of secondary rings decreases much faster for spherical vortices (∝1/*r*^2^) than for cylindrical vortices (∝1/*r*). Since radiation pressure is proportional to the intensity of the beam, it means that the selectivity is greatly enhanced by the axial focusing of the beam compared to cylindrical vortices. Thus, the 3D focalization of the energy is a major advantage for selective manipulation of particles. The slight differences between the experimentally measured field and the spherical Bessel function can be explained by the fact that we do not literally synthesize a spherical Bessel vortex. The phase of our signal matches the one of a spherical vortex but not the radial evolution of the amplitude.

**Fig. 3:**
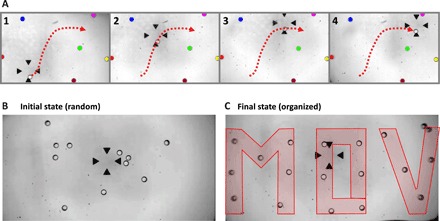
Microparticles’ selective displacement in a standard microscopy environment. (**A**) Selective manipulation of a polystyrene particle having a radius of 75 ± 2 μm with the 4.4-MHz selective acoustical tweezers based on Archimedes-Fermat spirals. This figure shows that only the particle trapped at the center of the vortex (located just above the lowest arrow, as shown in movie S3) is moved, while the other particles remain still. The particles at rest have been colored to improve the readability of the figure (see also movie S2). (**B** and **C**) Patterning of 18 polystyrene particles with a radius of 75 ± 2 μm into prescribed position to form the letters M, O, and V (moving object with vortices). (B) Randomly dispersed particles (initial state). (C) Organized particles (final state).

To demonstrate the selectivity of this acoustical tweezer, i.e., its ability to pick up one particle and move it independently of its neighbors, we dispersed some monodisperse polystyrene particles with a radius of 75 ± 2 μm inside a microfluidic chamber with a height of 300 μm and then picked one particle and moved it around between the other particles. Polystyrene particles were chosen for this demonstration, owing to their weak density and compressibility contrast with the surrounding liquid. As demonstrated by Baresch *et al*. (*4*), the trapping force exerted on solid particles by a first-order Bessel beam strongly relies on the density and/or compressibility contrast: The weaker this contrast, the weaker the trapping force. The power of our beam was chosen to produce a sufficient force to move the target particle while keeping the second ring magnitude sufficiently weak not to be able to trap particles. Figure 3A and movie S2 show that, as long as the distance between the transported particle and the other particles remains larger than the radius of the first bright ring *r*_1_, only the trapped particle moves. As might be expected, when the first ring touches a particle located outside it, the particle is pushed away since the rings are repulsive. Note that, in this movie, the center of the vortex is located at the tip of the bottom arrow where the particle is trapped (as demonstrated by the scan of the acoustic field; movie S3). Two particles can thus be individually selected if their distance exceeds the radius of the first bright ring, which can be estimated from the calculation of the first maximum of the spherical Bessel function. Here, this distance is substantially larger than the radius of the particle (3.3 times), since we originally designed our tweezers to also have 3D trapping capabilities. This ability requires a first ring over particle radius ratio (*r*_1_/*a*) of this order, as demonstrated by Baresch *et al*. (*5*), for similar aperture and particles. Too large particles would be pushed away from the trap with a one-sided acoustical tweezer. Nevertheless, for the lateral manipulation only, the optimal trapping force is achieved for a ratio *r*_1_/*a* equal to unity. In this limit, it would be possible to discriminate particles that are almost in contact. We additionally demonstrated the tweezers’ ability to precisely position a set of 18 polystyrene particles with a radius of 75 ± 2 μm in a prescribed pattern: MOV (moving objects with vortices), starting from randomly distributed particles (see Fig. 3, B and C).

## DISCUSSION

Acoustical tweezers have long faced a trade-off between selectivity and miniaturization/integrability. These restrictions have so far prevented the development of many applications in microfluidics and microbiology. Here, these limitations are overcome by unifying the physical principles of (i) acoustic trapping with focalized vortices, (ii) holographic wave synthesis with IDTs, and (iii) Fresnel lenses inside a single compact transparent miniaturized device. With this microsystem, we demonstrate the contactless manipulation of particles in a standard microscopy environment with state-of-the-art selectivity. Owing to the simplicity of the technology and its scalability to higher frequencies, this work paves the way toward individual manipulation and in situ assembly of physical and biological micro-objects. In conclusion, the tweezers designed in this work satisfy the prerequisite for 3D trapping (wave field topology and aperture). Nevertheless, the rigorous demonstration of real 3D trapping capabilities with a progressive wave would require getting rid of any spurious standing waves that might appear from wave reflections at the walls and thus should be performed in a large tank to simulate free space conditions. These conditions are orthogonal to the present experiments centered on selective trapping in a standard microscopy (and thus confined) environment. The demonstration of 3D trapping capabilities of Archimedes-Fermat tweezers would therefore constitute an interesting perspective to this work.

## Supplementary Material

http://advances.sciencemag.org/cgi/content/full/5/4/eaav1967/DC1

Download PDF

Movie S1

Movie S2

Movie S3

## SUPPLEMENTARY MATERIALS

Supplementary material for this article is available at http://advances.sciencemag.org/cgi/content/full/5/4/eaav1967/DC1 Fig. S1. Comparison of the shape of the electrodes obtained by approximated Eq. 2 and exact Eq. 1. Fig. S2. Image illustrating movie S1 showing an animation of the vortex measured experimentally with a UHF-120 Polytec laser interferometer. Fig. S3. Image illustrating movie S2 showing the selective manipulation of polystyrene particle having a radius of 75 ± 2 μm with the 4.4-MHz selective acoustical tweezers based on Archimedes-Fermat spirals. Fig. S4. Image illustrating movie S3 showing the vortex center located at the tip of the bottom arrow where the particle is trapped. Movie S1. Movie showing an animation of the vortex measured experimentally with a laser interferometer. Movie S2. Movie showing the selective manipulation of polystyrene particle having a radius of 75 ± 2 μm with the 4.4-MHz selective acoustical tweezers based on Archimedes-Fermat spirals. Movie S3. Movie showing the localization of the vortex core compared to the localization of the arrows.
