# Supplementary material for: Folding a focalized acoustical vortex on a flat holographic transducer: Miniaturized selective acoustical tweezers
Source: Sci Adv. 2019 Apr 12;5(4):eaav1967. doi: 10.1126/sciadv.aav1967 (PMC6461452; doi:10.1126/sciadv.aav1967)
Supplement: http://advances.sciencemag.org/cgi/content/full/5/4/eaav1967/DC1 [file supp_5_4_eaav1967__index.html]

Science Advances | Science Advances

## Supplementary Materials

**The PDF file includes:**

- Fig. S1. Comparison of the shape of the electrodes obtained by approximated Eq. 2 and exact Eq. 1.
- Fig. S2. Image illustrating movie S1 showing an animation of the vortex measured experimentally with a UHF-120 Polytec laser interferometer.
- Fig. S3. Image illustrating movie S2 showing the selective manipulation of polystyrene particle having a radius of 75 ± 2 μm with the 4.4-MHz selective acoustical tweezers based on Archimedes-Fermat spirals.
- Fig. S4. Image illustrating movie S3 showing the vortex center located at the tip of the bottom arrow where the particle is trapped.

Download PDF

**Other Supplementary Material for this manuscript includes the following:**

- Movie S1 (.mp4 format). Movie showing an animation of the vortex measured experimentally with a laser interferometer.
- Movie S2 (.mp4 format). Movie showing the selective manipulation of polystyrene particle having a radius of 75 ± 2 μm with the 4.4-MHz selective acoustical tweezers based on Archimedes-Fermat spirals.
- Movie S3 (.mp4 format). Movie showing the localization of the vortex core compared to the localization of the arrows.

**Files in this Data Supplement:**

- Adobe PDF - aav1967\_SM.pdf
