# Supplementary material for: Folding a focalized acoustical vortex on a flat holographic transducer: Miniaturized selective acoustical tweezers
Source: Sci Adv. 2019 Apr 12;5(4):eaav1967. doi: 10.1126/sciadv.aav1967 (PMC6461452; doi:10.1126/sciadv.aav1967)
Supplement: Download PDF [file aav1967_SM.pdf]

Science Advances
AAAS

advances.sciencemag.org/cgi/content/full/5/4/eaav1967/DC1

Supplementary Materials for

Folding a focalized acoustical vortex on a flat holographic transducer: Miniaturized selective acoustical tweezers

Michael Baudoin*, Jean-Claude Gerbedoen, Antoine Riaud, Olivier Bou Matar, Nikolay Smagin, Jean-Louis Thomas

*Corresponding author. Email: michael.baudoin@univ-lille1.fr

Published 12 April 2019, *Sci. Adv.* **5**, eaav1967 (2019)
DOI: 10.1126/sciadv.aav1967

The PDF file includes:

Fig. S1. Comparison of the shape of the electrodes obtained by approximated Eq. 2 and exact Eq. 1.
Fig. S2. Image illustrating movie S1 showing an animation of the vortex measured experimentally with a UHF-120 Polytec laser interferometer.
Fig. S3. Image illustrating movie S2 showing the selective manipulation of polystyrene particle having a radius of 75 ± 2 μm with the 4.4-MHz selective acoustical tweezers based on Archimedes-Fermat spirals.
Fig. S4. Image illustrating movie S3 showing the vortex center located at the tip of the bottom arrow where the particle is trapped.

Other Supplementary Material for this manuscript includes the following:

(available at advances.sciencemag.org/cgi/content/full/5/4/eaav1967/DC1)

Movie S1 (.mp4 format). Movie showing an animation of the vortex measured experimentally with a laser interferometer.
Movie S2 (.mp4 format). Movie showing the selective manipulation of polystyrene particle having a radius of 75 ± 2 μm with the 4.4-MHz selective acoustical tweezers based on Archimedes-Fermat spirals.
Movie S3 (.mp4 format). Movie showing the localization of the vortex core compared to the localization of the arrows.

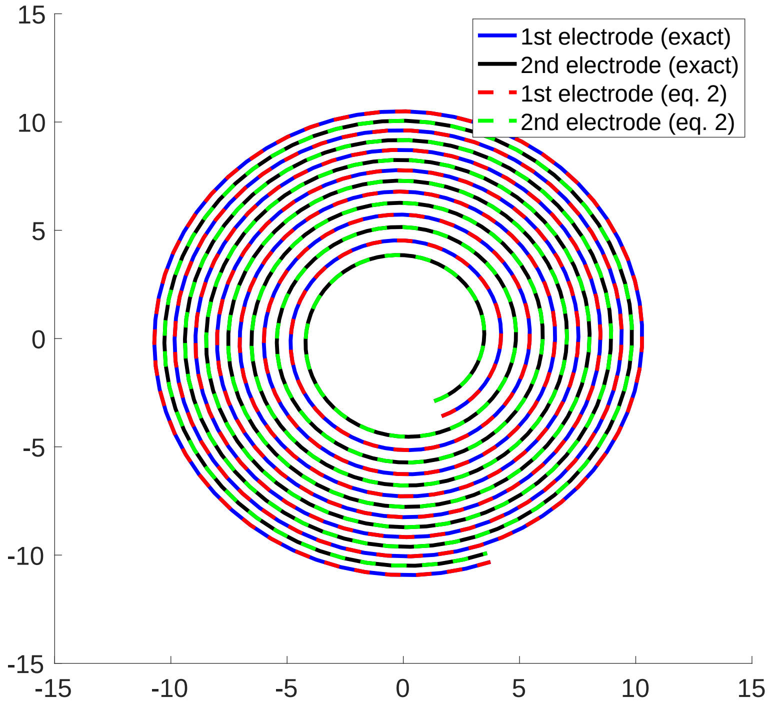

Fig. S1. Comparison of the shape of the electrodes obtained by approximated Eq. 2 and exact Eq. 1. Blue solid line: shape of the electrodes obtained with exact equation (1). Black solid line: shape of the second electrode (phase shift: π) obtained with exact equation (1). Red dashed line: shape of the electrodes obtained with approximated equation (2). Green dashed line: shape of the second electrode (phase shift: π) obtained with approximation equation (2). This figure shows no discernible difference between exact and approximated equations.

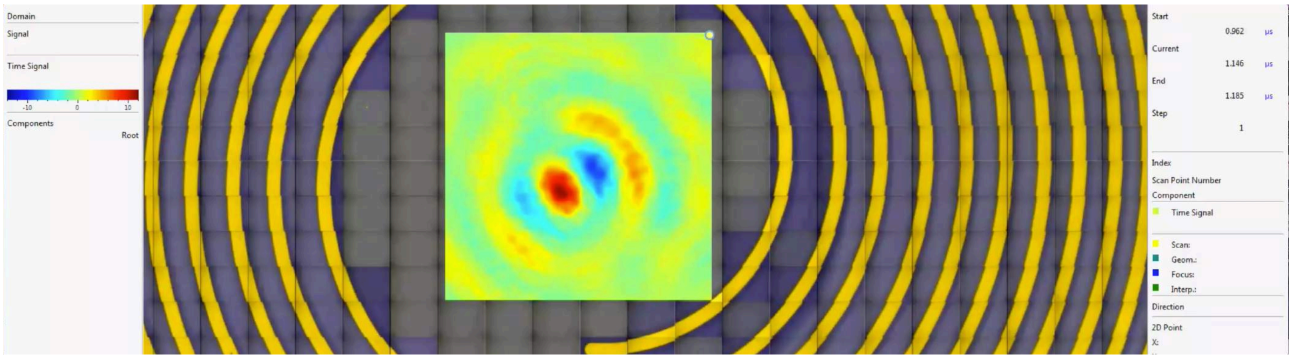

Fig. S2. Image illustrating movie S1 showing an animation of the vortex measured experimentally with a UHF-120 Polytec laser interferometer. Color correspond to the amplitude of the normal displacement at the surface of the coverslip. In the colorbar, the magnitude of the normal displacements are given in nanometers.

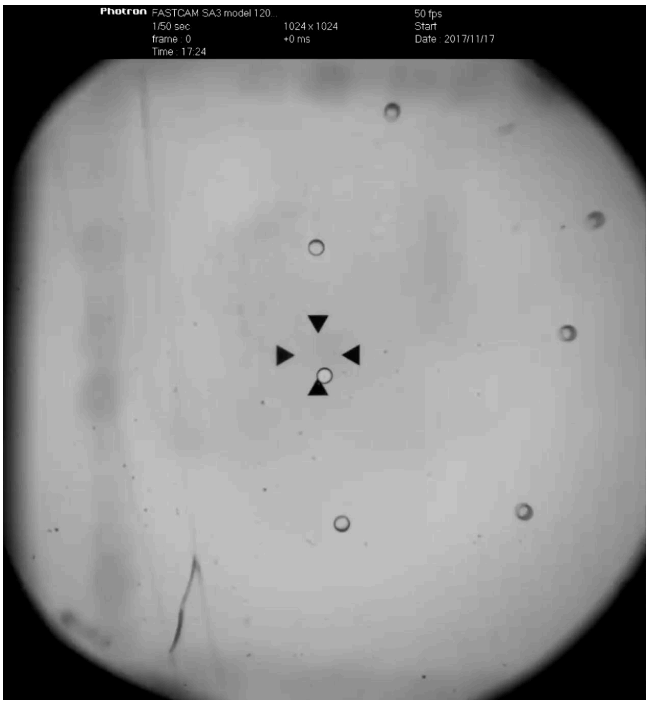

Fig. S3. Image illustrating movie S2 showing the selective manipulation of polystyrene particle having a radius of 75 ± 2 µm with the 4.4-MHz selective acoustical tweezers based on Archimedes-Fermat spirals. This figure shows that only the particle trapped at the center of the vortex (located just above the lowest arrow as shown on movie M3 in SM) is moved while the other particles remain still. The datas correspond to Fig.3.

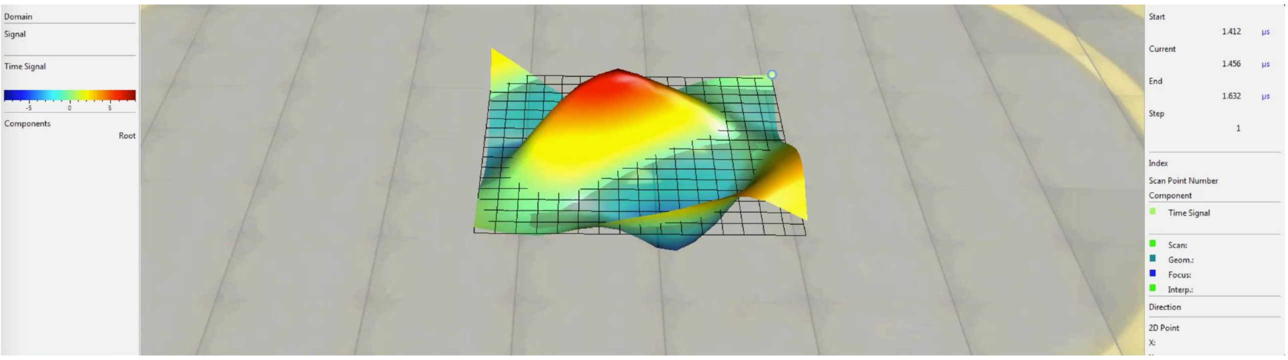

Fig. S4. Image illustrating movie S3 showing the vortex center located at the tip of the bottom arrow where the particle is trapped.
